# A Woman on "Woman at Home"

**Published:** 1887-11-19

**Authors:** 


### A Woman on "Woman at Home."

A "Mistress and a Mother" writes: The "short and sensible" (if not sweet) sermon preached from your pulpit this last week finds an echo in the heart of at least one married woman among your readers. The truths, "wholesome if not toothsome," which it so decidedly and earnestly sets forth have been in my mind for a long while, and I am so glad that someone has put them into words, which I hope may be read by many of my sisters, married and single. The married, alas! may read only to sigh over the unhappy truth of your words; but I trust that with the single sisters the perusal of them may prevent those words from ever being true in their particular case.

As you so pithily put it—

Firstly. Health is greatly dependent on that assembly of circumstances which makes up the complex idea of "home." For most men life is made up of work and home, business and domestic concerns!

Secondly. Woman also is a specialist, and her duties for the most part lie at home. Whether for weal or for woe, home is woman's kingdom! Now, anyone making use of the common sense with which God has gifted them, must at once cap those two heads with a third.

"Therefore woman, who is to reign in that home which is so greatly to make or to mar the body and soul and spirit of the man who lives in it, should be thoroughly trained and educated for her duties before entering upon them."

And yet (to our shame be it spoken) many married women (myself among them) enter upon their life-work in the home God gives to them with no more knowledge of making a pudding than that required for the "mud-pies" of childhood's games. No more idea of caring for a tender little baby than that with which they alternately hugged and neglected their dollies! They promise at the altar to cherish "in sickness" as well as health the husband whom they really love, but when the "sickness" comes their knowledge of a nurse's duties consists only in "giving a pill." They undertake to manage from one to five (or more) servants, but their servants soon learn to manage them, not, perhaps, from want of will, but from an utter absence of the experience required.

To sensible women all this knowledge comes in time, and is slowly acquired by painful experience after marriage, so that at the end of a few years the wife, mother, and mistress finds herself equal to her duties as queen of the home. But, oh! the miserable failures, the many heartburnings, the bitter tears of those "few years" who shall recount?

A young girl is carefully and virtuously brought up by a tender mother, who, knowing the bent of her daughter's mind, says, lovingly, *but not wisely*, "Enjoy your books and your amusements now, darling; there is very little time for these in after-life, *especially* if you marry."

In time the girl gives her heart, with all its wealth of strong romantic love, to the one whom she thinks God has chosen as her life companion. Then a vague questioning arises as to her fitness to rule in his home. But the tender mother assures her, "It will all come by experience, just as it did to her." But what an experience! Soon after marriage, the husband, who was so proud at first of his little wife's cleverness, finds that somehow it is not very practical. The bills mount up. The one middle-aged general servant is obstinate, and will do things "her own way," which does not happen to be "the way" of her master and mistress; but the latter seems unable to teach it to her, and the master wonders if the secret of her failure lies in the undertoned grumble which he overheard from the kitchen: "Missus is as hignorant as a child about heverythink!"

After a time God sends to the little home a sweet, helpless babe, claiming a mother's tenderest care. Poor little baby! No wonder you cry so often and so long! Mother is only learning by experience what kind of care a baby requires, but it is not a "pleasant experience" for *you*. She loves you so much; but she "never saw a baby washed before," and "never held a baby in her arms" till she held you!

Time passes on. The care of a larger house and of several servants suddenly devolves on our heroine, who is now always in a state of "worry." And no wonder. The servants are continually quarrelling about their work, and she is not quite certain as to what work belongs to which!

Meantime other little ones have come with quick-following footsteps to nestle beneath the parent wing. And sorely strained and terribly tried is that mother and mistress with the daily routine of work and worry. Outside claims connected with the new and widened sphere in which her husband moves now also take up her time and attention. What wonder that the little ones, left more than formerly to the care of nurses not stricly enough overlooked, are attacked with sickness. The mother has had "no experience" in sick nursing, and fancies it is only "a trifling ailment"; and only when the sweet child-prattle, the little pattering footfall, the love-look at mother, the pretty baby ways are all stilled and silenced and stayed for ever in this world by the icy hand of death, does the poor mother awake to the awful fact that mainly through her ignorance (humanly speaking) a lifelong sorrow has come to her heart and home.

And following the death of the little ones, comes another sorrow in its train—the lessening of love between the husband and wife; for all true love is founded on trust, and since the husband's confidence in his wife's wisdom has had so severe a shaking, his love for her suffers also.

"How differently my mother managed her household and brought up her children," is continually, and often not kindly, brought before his wife's notice, until with secret tears of bitter humiliation she feels that the mother-in-law, to whom she had always thought herself so superior "in mind and manner" has an advantage over her in her husband's esteem and in her practical knowledge of the common duties of a woman's life.

There, my dear doctor, though my pen and words may seem to have run on lightly, I have written with a sorrowing spirit the sad story of one married woman's experience—and of how many more?

But with all due deference to your "mental superiority" as a man, there is one point in your sermon on which I beg to differ, viz., that matters being thus proves the "mental inferiority" of *women*. Rather does it spring from a virtue exaggerated, a desire "to please" the men. You take it as an accepted fact that the majority of women will marry and rule a home of their own. Granted, for argument's sake. The majority of young women will therefore take up naturally the position which most pleases their future husbands. Now I ask you, Is it the useful, practical, educated girl who knows how to bake a loaf, to write a thorough letter, to nurse an invalid on hospital lines, to make up accounts, to reign and rule among children and servants as a God-fearing matron—is this the sort of girl who is most sought after among the young men of the present day? Nay, my dear doctor, I have recommended such girls to young men under my own roof, and have met with the answer, "Oh! no doubt she is an excellent young lady, but then she has no figure; or, she has such a queer nose!" And so the girl with a pretty face, who can rattle a tune on the piano, sing a nice little solo, play a good game of tennis, and flirt pleasantly, is chosen in preference to the sensible girl, who doubtless is equally good-looking, but does not find time to make dressing daintily, braiding her hair becomingly, and squeezing her waist and feet and hands into coquettish coverings, her first and greatest work in life.

True! I was consulted lately by a bachelor, who meditated matrimony, as to the capabilities of a young lady friend, in such questions as these: Did she understand cooking, as well as French? Was she domesticated, as well as educated? And was she healthy? Indeed, Could I tell him of what her father died? But then, he was a bachelor of forty-seven! No one could expect such foresight and wisdom from one of twenty-seven—unless he were a doctor!

By all means preach from your pulpit to the young ladies of our land till we see a happy change in the education of the day; but preach likewise to our young men till they demand for their future wives an educated mind before a pretty face; a tender nurse before an elegant waltzer; a clever cook before a capital tennis-player; and a girl who studies her Bible in preference to one who lolls over "Ouida's" latest. This will hasten a happy reform sooner than aught else.

If you consider it will in any way further this object, please publish this testimony of one who for seven years has been a mistress and a mother.

